# Advances and Challenges in Targeted Therapy and Its Combination Strategies for Leukemia

**DOI:** 10.3390/biomedicines13071652

**Published:** 2025-07-07

**Authors:** Zhiyuan Zhong, Ran Yao, Yifei Duan, Cheng Ouyang, Zefan Du, Lindi Li, Hailin Zou, Yong Liu, Hongman Xue, Liang Li, Chun Chen

**Affiliations:** 1 Pediatric Hematology Laboratory, Division of Hematology/Oncology, Department of Pediatrics, The Seventh Affiliated Hospital of Sun Yat-Sen University, Shenzhen 518107, China; zhongzhiyuan@sysush.com (Z.Z.); dyf0824@126.com (Y.D.); ouycheng@mail2.sysu.edu.cn (C.O.); duzf3@mail2.sysu.edu.cn (Z.D.); lild9@mail2.sysu.edu.cn (L.L.); liuy995@mail2.sysu.edu.cn (Y.L.); xuehongman@sysush.com (H.X.); 2 Scientific Research Center, The Seventh Affiliated Hospital of Sun Yat-Sen University, Shenzhen 518107, China; yaoran1@sysush.com (R.Y.); zouhlin@mail2.sysu.edu.cn (H.Z.)

**Keywords:** leukemia, targeted therapy, immunotherapy drugs, combination therapy

## Abstract

Leukemia is a group of hematological malignancies with a complex pathogenesis and diverse clinical manifestations. Although traditional treatments such as chemotherapy, radiotherapy, and hematopoietic stem cell transplantation have improved patient outcomes, their efficacy is often limited by non-specificity, drug resistance, and relapse. In recent years, targeted therapy has emerged as a major breakthrough, offering new opportunities for precision medicine in leukemia. The development of molecularly targeted agents has significantly advanced our ability to treat specific leukemia subtypes. However, challenges such as resistance to targeted drugs, adverse effects, and tumor heterogeneity remain significant obstacles. As a result, treatment strategies are shifting from single-agent chemotherapy toward combination therapies that integrate targeted agents, aiming to enhance therapeutic efficacy and reduce the likelihood of resistance. This review summarizes the current research landscape, clinical applications, and limitations of targeted therapies in leukemia, with a focus on recent progress in combination treatment strategies and ongoing clinical trials.

## 1. Introduction

Leukemia is a class of malignant hematological diseases caused by the abnormal clonal proliferation of hematopoietic stem cells. Its pathogenesis is complex, involving multiple factors such as genetic mutations, environmental exposure, infection, and endocrine factors [1,2,3]. The typical pathological feature is the accumulation of a large number of leukemia cells in the bone marrow and hematopoietic tissues, inhibiting normal hematopoietic function, and it can also infiltrate other organs, leading to anemia, bleeding, infection, and multiple organ dysfunction [4,5,6]. According to the differentiation stage and pathological characteristics of leukemia cells, leukemia can be divided into types such as acute lymphoblastic leukemia (ALL), acute myeloid leukemia (AML), chronic lymphocytic leukemia (CLL), and chronic myeloid leukemia (CML). Among them, ALL is common in children and adolescents, with the peak age of onset between 2 and 5 years old. AML is the most common type in adults, while the course of chronic leukemia is relatively slow but may transform into blast crisis [7,8]. According to statistics, in 2022, the global incidence and mortality of leukemia ranked 13th (486,777 cases) and 10th (305,033 cases), respectively, among all cancers, seriously threatening human health [9].

The traditional treatment methods for leukemia are mainly chemotherapy, but chemotherapy has many serious side effects, which may lead to treatment failure due to treatment-related death or drug resistance [10]. With the emergence of new treatment methods such as targeted therapy and immunotherapy, the treatment of leukemia has shifted from cytotoxic chemotherapy to personalized medical treatment [11]. Unlike traditional chemotherapy, targeted therapy can specifically interfere with target macromolecules related to carcinogenesis, rather than acting on all rapidly dividing cells. In addition, targeted therapy can effectively enhance the therapeutic effect of leukemia by limiting the occurrence of acquired resistance during treatment, improving the efficacy of individual components in cancer treatment mixtures, and reducing related side effects [12,13]. Although targeted drugs have achieved certain success in the treatment of leukemia, single-agent use has many limitations, such as resistance and a series of adverse reactions triggered during treatment, which seriously affect the patient’s quality of life and treatment compliance, thus affecting the therapeutic effect. Therefore, the treatment of leukemia has gradually shifted from single chemotherapy to targeted drug combination therapy, significantly improving the remission rate and survival rate of various types of leukemia [14,15,16].

In this review, we discuss the therapeutic effects and limitations of single-agent use of currently approved targeted drugs for leukemia treatment, such as tyrosine kinase inhibitors (TKIs) and FLT3 inhibitors. Subsequently, we further propose combination therapies in the forms of targeted drugs combined with chemotherapy drugs, internal combination of targeted drugs, targeted drugs combined with chimeric antigen receptor T-cell immunotherapy (CAR-T), and multi-drug combination as key strategies to enhance efficacy, hopefully bringing better treatment prospects for leukemia patients.

## 2. Targeted Therapy

Targeted therapy improves the precision and effectiveness of leukemia treatment by identifying and attacking specific molecular targets unique to leukemia cells, such as gene mutations or abnormal signaling pathways. Based on the mechanism of action and targets, targeted drugs can be divided into TKIs, FLT3 inhibitors, B-cell signaling pathway inhibitors, anti-apoptotic inhibitors, and immunotherapy drugs, etc.

### 2.1. Tyrosine Kinase Inhibitors

Tyrosine kinases play a key role in intracellular signal transduction. These enzymes regulate various cellular functions, such as growth, division, migration, and survival, by catalyzing the phosphorylation of tyrosine residues on specific proteins. Their abnormal activation can trigger tumor development, thus becoming important therapeutic targets. TKIs are small-molecule oral inhibitors that block the activity of tyrosine kinases by competitively inhibiting ATP binding at the catalytic tyrosine kinase binding site, thereby inhibiting carcinogenic signal transduction [17]. Currently, various TKIs such as Imatinib, Dasatinib, and Ponatinib have been developed, which can effectively target CML and Philadelphia chromosome-positive acute lymphoblastic leukemia (Ph+ALL) patients.

#### 2.1.1. Imatinib

Before the advent of targeted TKIs, the treatment of leukemia mainly relied on chemotherapy, radiotherapy, and hematopoietic stem cell transplantation (HSCT), but the overall effect was poor. For example, HSCT, as a potential curative method for early CML, is limited by various factors, including whether the patient meets the transplant criteria, the risk of early treatment-related death, and long-term disability caused by chronic graft-versus-host disease [10,18]. In 1960, researchers discovered that the Philadelphia chromosome was the cause of CML development. The ABL gene on chromosome 9 translocated with the BCR gene on chromosome 22, forming the BCR-ABL fusion gene. The tyrosine kinase activity encoded by this gene is uncontrolled, leading to uncontrolled cell division [19]. By the late 1990s, Nick Lydon of Novartis synthesized a compound called CGP57148, the predecessor of Imatinib, which was promoted for CML treatment by oncologist Brian Druker at the Dana-Farber Cancer Institute. The drug underwent its first clinical trial in 1998 and received FDA approval in May 2001, officially becoming a first-line treatment for CML [20,21]. The discovery and application of Imatinib was a major breakthrough in leukemia treatment, opening a new chapter in targeted therapy for leukemia.

**Figure 1:**
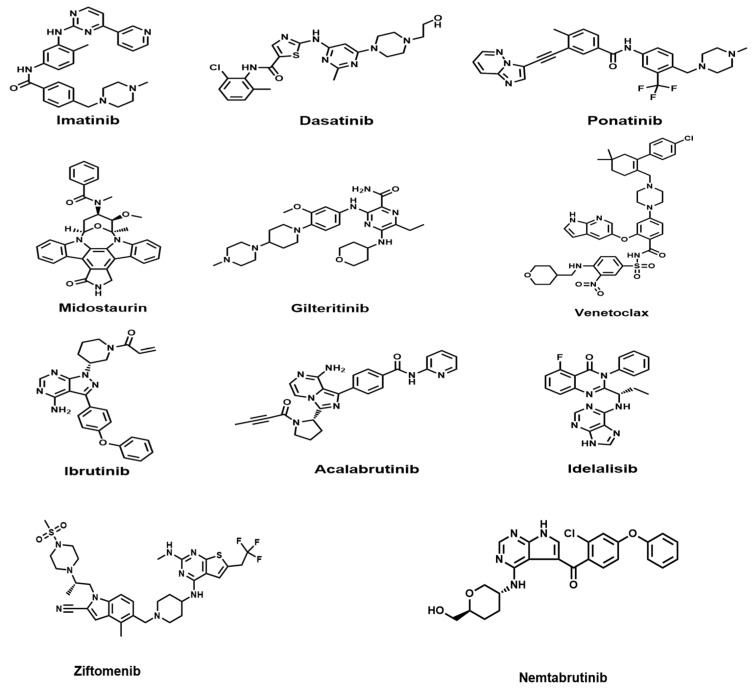
Chemical structural formulae of Imatinib, Dasatinib, Ponatinib, Midostaurin, Gilteritinib, Ibrutinib, Acalabrutinib, Idelalisib, Venetoclax, Ziftomenib, Nemtabrutinib.

**Figure 2:**
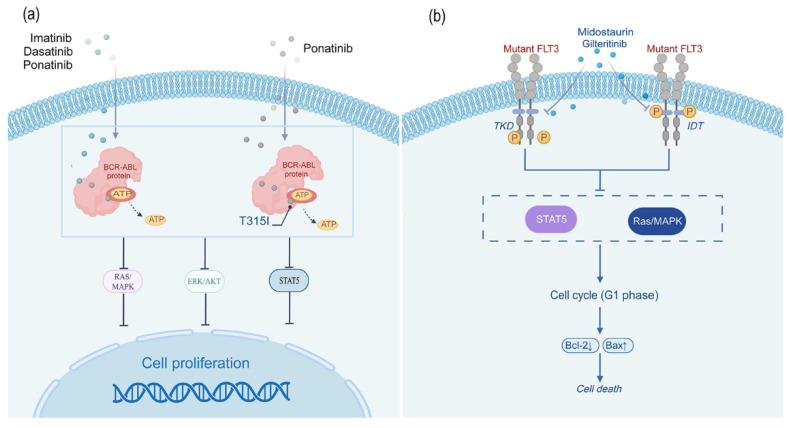
Mechanism of action diagram for Imatinib/Dasatinib/Ponatinib and Midostaurin/Gilteritinib, etc. (**a**) Imatinib/Dasatinib/Ponatinib, etc., selectively inhibits the ATP-binding site of the BCR-ABL fusion protein (Ponatinib can be adapted to T315I mutants that are resistant to other TKIs), blocking its tyrosine kinase activity, inhibiting signaling pathways such as RAS/MAPK, PI3K/AKT, STAT, etc., and blocking leukemia cell proliferation and survival. (**b**) Midostaurin/Gilteritinib reduces leukemia cell proliferation and induces apoptosis by binding highly selectively to the ATP-binding site of FLT3 mutant proteins, inhibiting their kinase activity, and blocking the phosphorylation of downstream signaling pathways (e.g., STAT5, Ras/MAPK).

As the first-generation TKI, Imatinib (Figure 1) effectively treats CML and Ph+ALL patients by binding to the catalytic domain of the BCR-ABL protein, preventing ATP binding, thereby inhibiting the phosphorylation of tyrosine residues on various substrates, ultimately preventing cancer cell proliferation (Figure 2) [14,22]. Research shows that the 10-year survival rate for Imatinib treatment of CML is 83.3% [22]. It can achieve durable cytogenetic and molecular remission, significantly improving the survival rate of most patients [23]. Clinical trials such as STIM and TWISTER showed that approximately 39–45% of patients with sustained deep molecular response (DMR) can maintain treatment-free remission (TFR) for 3 years or longer after receiving Imatinib treatment [24]. After 6 years of Imatinib treatment, the proportion of patients reaching TFR can reach 21.6% [25]. Moreover, drug discontinuation does not lead to acquired resistance to Imatinib, nor does it trigger further safety issues. Ultimately, nearly 10% of patients can completely stop medication [24,26].

Although Imatinib has a certain therapeutic effect on leukemia, mutations in the BCR-ABL gene, especially the T315I mutation, can trigger drug resistance, limiting its clinical application. Research shows that about 25–30% of CML patients develop resistance to Imatinib [27]. In addition, Imatinib may cause mild to moderate adverse reactions such as superficial edema, pigmentation, and rash during treatment. Keshavamurthy Vinay et al. studied 438 patients and found that these patients took Imatinib for an average of 1820 days. Among them, 53.9% developed melasma-like pigmentation; 18.5% developed periorbital edema; 16% developed oral lichenoid reactions; 9.6% developed skin hypopigmentation, and 2.7% developed bullous pemphigoid [28]. Therefore, it is necessary to develop more effective drugs to reduce drug toxicity and improve the treatment efficiency of leukemia.

#### 2.1.2. Dasatinib

Dasatinib (Figure 1), as a second-generation TKI, is more potent against BCR-ABL activity compared to Imatinib. It can effectively address a wider range of mutation situations, such as the E255K and G250E mutations within the phosphate-binding loop of the ATP-binding pocket, playing a key role in transforming CML into a manageable disease [15]. The 10-year follow-up results of the IRIS trial showed that for CML treatment, its 5-year overall survival (OS) rate reached 96%, treatment failure-free survival reached 95%. At the same time, the 5-year cumulative major molecular response (MMR) rate was 89%, and the MR4.5 rate (i.e., the ratio of abnormal gene copies decreasing by 4.5 logarithms) reached 79%. Among patients who achieved MR4.5, those who maintained this status for ≥2 years, 11% voluntarily chose to stop medication, and the 2-year TFR rate after discontinuation was 58% [16]. This indicates that Dasatinib, as a first-line treatment drug, has good long-term safety and efficacy in newly diagnosed CML and Ph+ALL.

Although Dasatinib is effective for Imatinib-resistant/intolerant CML and Ph+ALL, it may cause adverse reactions such as cardiovascular side effects, pulmonary arterial hypertension, pleural effusion, and bleeding in clinical applications. In addition, kinase resistance mutations, including T315I, disrupt drug binding, leading to increased Dasatinib resistance [29]. Therefore, continuous research and development of new treatment methods are needed to improve the usability of the drug.

#### 2.1.3. Ponatinib

As a third-generation TKI, Ponatinib (Figure 1) is more effective than Dasatinib for CML and Ph+ALL patients with resistance or intolerance, especially those with the T315I mutation (Figure 2) [30]. In 2012, the drug received FDA approval. In October 2013, due to serious safety concerns, Ponatinib was voluntarily withdrawn from the market by its manufacturer. In December of the same year, the drug was reintroduced under revised warnings and precautions. Its indication was limited to patients with the T315I mutation or those who are not suitable for other TKI therapies [31].

According to long-term follow-up results of Ponatinib treatment for CML patients, the 10-year OS rate is 90%, and the 2-year event-free survival (EFS) rate is 97%. After 6 months of treatment, 96% of patients achieved complete cytogenetic response, 80% achieved MMR, 61% achieved MR4 (leukemia cells decreased by 4 logarithms, residual count less than 0.01%), and 46% achieved MR4.5. At the 12-month mark, 94% of patients achieved complete cytogenetic response, 81% achieved MMR, and the MR4 rate sustained for over 5 years was 51%. More importantly, no disease progression to accelerated phase or blast crisis occurred during the treatment period. However, 28% of patients discontinued medication due to cardiovascular toxicity [32].

The main adverse reactions of Ponatinib are arterial occlusive events, hypertension, gastrointestinal reactions, etc. Due to the increased risk of arterial occlusive events and other serious toxicities potentially induced by Ponatinib, it is currently not suitable as a first-line treatment option [32,33].

In general, the application of TKIs has brought significant improvements in treatment outcomes for CML patients. For example, the development of Imatinib has reduced the annual mortality rate of CML from 10–20% to 1–2% [34]. However, the resistance issues and other adverse reactions caused by long-term use of TKIs prompt us to continuously explore new targets.

### 2.2. FLT3 Inhibitors

In recent years, studies have found that mutations in the FLT3 gene are relatively common in AML, accounting for about 30% of AML cases [35]. FLT3 mutations lead to abnormal activation of the FLT3 receptor, which continuously activates downstream signaling pathways such as Ras/MAPK/, STAT5, PI3K/Akt/mTOR, inhibits cell apoptosis, and promotes the proliferation and survival of leukemia cells [36]. FLT3 inhibitors are a class of targeted therapy drugs for FLT3-mutated AML that have been approved for clinical targeted therapy. According to the mechanism of action and targets, FLT3 inhibitors can be divided into first-generation and second-generation FLT3 inhibitors. These drugs inhibit the abnormal activation of the FLT3 receptor, prevent the proliferation and survival of leukemia cells, and significantly improve the therapeutic effect and survival rate of AML patients.

#### 2.2.1. Midostaurin

Midostaurin (Figure 1) is an oral multi-kinase small-molecule inhibitor and the first-generation FLT3 inhibitor. It mainly inhibits the tyrosine kinase activity of the FLT3 receptor, blocks its downstream signaling pathways, thereby inhibiting the proliferation and survival of leukemia cells and inducing apoptosis (Figure 2). It was approved by the FDA in 2017 for the treatment of AML and is also the first approved FLT3 inhibitor in the United States and Europe [37].

Although Midostaurin has been approved for adult FLT3-mutated AML, its effect is suboptimal for relapsed or refractory pediatric acute leukemia patients. A phase 1/2 clinical trial showed that compared to ALL patients with KMT2A rearrangement, FLT3-mutated AML patients showed more significant advantages in overall response rate (55.5% vs. 23.1%) and overall survival (3.7 months vs. 1.4 months) [38]. In addition, long-term use of Midostaurin is associated with adverse reactions such as low blood cell counts (neutropenia, anemia, thrombocytopenia), severe allergic reactions, and liver function problems [39].

#### 2.2.2. Gilteritinib

Gilteritinib (Figure 1), as a second-generation FLT3 inhibitor, is an oral small-molecule drug. It has important therapeutic significance for AML patients carrying FLT3 internal tandem duplication mutations (FLT3-ITD) and has been approved for the treatment of relapsed/refractory FLT3-mutated acute myeloid leukemia [40]. Compared with first-generation FLT3 inhibitor therapy, Gilteritinib can effectively inhibit multiple secondary resistance mutations, thereby overcoming resistance issues and more effectively improving the survival rate and response rate of AML patients. The 2-year follow-up results of the ADMIRAL trial showed that among 247 patients in the Gilteritinib group, 26 patients survived for 2 years or more without relapse. Among them, 18 patients received HSCT, and 16 patients continued to use this drug for maintenance therapy after HSCT. During the maintenance treatment phase, Gilteritinib also showed good efficacy, enabling sustained remission status. This indicates that Gilteritinib has good efficacy and safety for FLT3-mutated AML, providing a better choice for the subsequent treatment of AML patients, especially when formulating long-term treatment plans [41].

The main side effect of Gilteritinib monotherapy is the development of acquired drug resistance. This resistance is closely related to the reactivation of the PI3K/AKT and RAS/MEK pathways. The activation of these pathways promotes the survival and proliferation of tumor cells, thereby increasing drug resistance [42]. This indicates that the management of drug resistance needs to be considered in subsequent treatment.

### 2.3. B-Cell Signaling Pathway Inhibitors

B-cell signaling pathway inhibitors are a class of targeted therapy drugs targeting key molecules or kinases in the B-cell signaling pathway. These drugs inhibit key components of the B-cell signaling pathway, prevent the proliferation and survival of leukemia cells, and significantly improve the therapeutic effect and survival rate of CLL and certain types of ALL patients. The main B-cell signaling pathway inhibitors include Bruton’s tyrosine kinase (BTK) inhibitors and PI3Kδ inhibitors, etc. [43].

#### 2.3.1. BTK Kinase Inhibitors

BTK is a protein that plays a key role in malignant tumor signal transduction. BTK participates in the differentiation and proliferation of B cells by binding to the B-cell receptor (BCR) [44]. This characteristic makes BTK a hot drug target for treating B-cell-related diseases. BTK inhibitors inhibit its tyrosine kinase activity by binding to the active site of BTK. This inhibition prevents the phosphorylation of downstream signaling molecules by BTK, thereby blocking the BCR signaling pathway transmission [45]. In recent years, BTK inhibitors (such as Ibrutinib, Acalabrutinib) have achieved significant success in the treatment of CLL, greatly improving the prognosis of patients.

##### Ibrutinib

**Figure 3:**
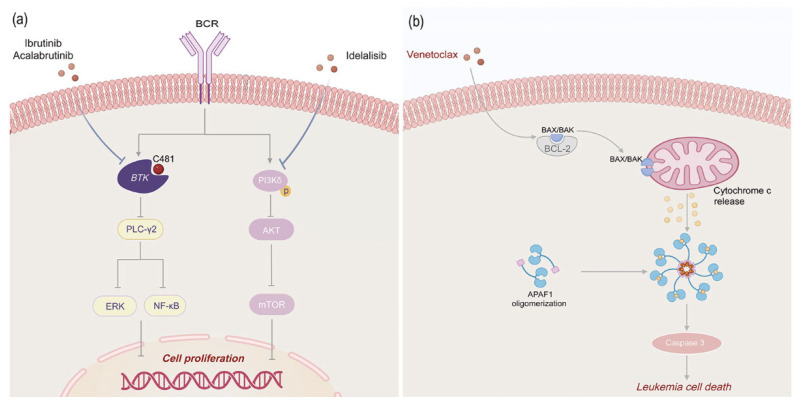
Mechanism of action diagram for Ibrutinib/Acalabrutinib/Idelalisib and Venetoclax. (**a**) Ibrutinib/Acalabrutinib covalently binds to Cys-481 near the ATP-binding domain of BTK, blocking BTK activation and inhibiting the BCR signaling pathway. Inhibition of BTK leads to blockage of activation of downstream signaling molecules such as tour ERK1/2, PI3K, and NF-κB, which inhibits leukemic B cell proliferation and survival. Idelalisib binds to the ATP binding site of PI3Kδ, inhibits the activity of PI3Kδ, blocks the BCR signaling pathway, which in turn leads to the blockage of downstream signaling pathways such as AKT, MAPK, NF-κB, etc., and inhibits the proliferation and survival of leukemic B cells. (**b**) Venetoclax replaces and releases pro-apoptotic proteins (e.g., BAX, BAK) by directly binding to Bcl-2 proteins, which initiates an apoptotic cascade that leads to the formation of a pore in the outer mitochondrial membrane, the release of cytochromes, and the further activation of caspase-3/7, which leads to the apoptotic death of malignant cells.

Ibrutinib (Figure 1) is the first BTK inhibitor approved for clinical use. It was approved by the FDA in 2016 for the initial treatment of CLL and is currently approved for patients with untreated, relapsed, or refractory disease, including patients with del(17p). PLCγ2 is a key downstream target of BTK. BTK inhibitors reduce their activity by preventing the phosphorylation of PLCγ2, thereby inhibiting the release of intracellular calcium ions and the hydrolysis of cell membrane phospholipids (Figure 3).

Follow-up results of an international multicenter phase III clinical trial showed that among elderly CLL patients, especially those with TP53 mutation, 11q deletion, and IGHV mutation, compared with traditional chlorambucil treatment plan, oral Ibrutinib treatment plan can achieve an overall response rate (ORR) of up to 92%, and its progression-free survival (PFS) (70% vs. 12%) and OS rate (83% vs. 68%) were significantly higher than the chlorambucil group [46].

Although Ibrutinib shows significant survival advantages in long-term studies, its side effects, such as atrial fibrillation and hypertension, lead to a higher treatment discontinuation rate. Research shows that among the selected 43 CLL patients, the cumulative incidence of atrial fibrillation was 30.0%, and the 5-year expected atrial fibrillation rate reached 31.5% [47]. Therefore, rational selection of targeted drugs and conducting combination therapy are crucial during the treatment process.

##### Acalabrutinib

Acalabrutinib (Figure 1) is a second-generation BTK inhibitor developed by AstraZeneca, characterized by high selectivity and potent effects. It has a shorter half-life and can be safely administered twice daily. This dosing method results in a longer remission period [48]. Compared with Ibrutinib, Acalabrutinib has similar efficacy in treating CLL, but relatively fewer toxic side effects [49]. In a phase I/II clinical study targeting relapsed/refractory (R/R)-CLL patients, Acalabrutinib showed good safety and efficacy, with an ORR of 94%. At 45 months, the estimated PFS value was 62% [50]. A phase 3 study further confirmed that compared with traditional treatment plans (Idelalisib plus Rituximab or Bendamustine plus Rituximab), Acalabrutinib monotherapy significantly improved the PFS of R/R-CLL patients and had acceptable safety. This drug was approved by the FDA in 2019 for the treatment of CLL [43].

The side effects of Acalabrutinib treatment for leukemia patients are similar to Ibrutinib, also prone to causing cardiovascular complications and bleeding adverse reactions. Research found that during Acalabrutinib treatment for CLL/Small Lymphocytic Lymphoma, the incidence of grade 3 and above adverse events reached 66%, including neutropenia (14%), pneumonia (11%), hypertension (7%), anemia (7%), and diarrhea (5%). The incidence rates of atrial fibrillation and major bleeding events were 7% and 5%, respectively. 56% of patients are still receiving treatment, with the main reasons for discontinuation being disease progression (21%) and adverse events (11%) [50]. Therefore, it is necessary to develop new targeted drugs or new treatment methods to reduce their toxicity.

#### 2.3.2. PI3Kδ Inhibitors

PI3Kδ inhibitors are a class of drugs that selectively target the PI3Kδ subtype. PI3Kδ is a member of the phosphatidylinositol 3-kinase (PI3K) family, mainly expressed in white blood cells, and is closely related to tumor development, immune regulation, and angiogenesis. PI3Kδ inhibitors inhibit tumor cell proliferation and survival by blocking PI3Kδ signaling, while also regulating the immune system [51].

Idelalisib (Figure 1) is a highly efficient and selective oral small-molecule PI3Kδ inhibitor. Its mechanism of action is to inhibit the PI3Kδ-dependent signaling pathway, reduce the activity of the AKT and mammalian target of rapamycin pathways, thereby inhibiting the activity of tumor cells (Figure 3) [45]. A phase I trial found that Idelalisib showed good safety and significant clinical efficacy in R/R-CLL patients. 81.5% of patients achieved lymph node response (50% reduction in the sum of the products of the perpendicular diameters (of measured lymph nodes) of the measured nodal lesions), the ORR reached 72%, and no dose-limiting toxicity was observed within the dose range. This drug received its first approval in the United States in July 2014 for the treatment of relapsed CLL [51].

Although Idelalisib has a good therapeutic effect on CLL, it can still trigger some adverse reactions during treatment, such as sepsis, pneumonia, diarrhea, and colitis, which limit its clinical application [52].

### 2.4. Anti-Apoptotic Inhibitors

Members of the anti-apoptotic B-cell lymphoma-2 (BCL-2) family are core regulatory factors in the apoptosis process. They are divided into three categories: anti-apoptotic proteins (such as BCL-2, BCL-XL, MCL-1), pro-apoptotic proteins (such as BAK, BAX), and pro-apoptotic proteins containing only the BH3 domain (such as BIM, BAD, NOXA). Among them, anti-apoptotic proteins inhibit apoptosis by binding to pro-apoptotic proteins and inhibiting their activity. BCL-2 inhibitors restore the natural pathway of apoptosis by mimicking the BH3 domain and binding to anti-apoptotic proteins (such as BCL-2), preventing them from interacting with other pro-apoptotic proteins [53].

Venetoclax (Figure 1), as the first selective BCL2 inhibitor, is also the first approved new anti-cancer drug (BH3 mimetic) for routine clinical treatment. Its mechanism of action is to directly target the BCL2 protein, reduce its inhibition of BAX and BAK proteins, thereby inducing CLL cell apoptosis (Figure 3) [54,55,56]. It is currently used for CLL and AML. However, long-term use may affect normal blood cells, leading to some adverse reactions, such as persistent cytopenias, clonal hematopoiesis, treatment-related myeloid neoplasms, and mutations in the BAX gene, thereby increasing resistance to Venetoclax treatment [53,57].

### 2.5. Immunotherapy Drugs

Immunotherapy drugs are a class of treatment methods that identify and attack leukemia cells by activating or enhancing the patient’s own immune system. According to the mechanism of action and targets, immunotherapy drugs can be divided into bispecific T-cell engagers (BiTEs) and monoclonal antibodies, etc. These drugs show significant efficacy and lower side effects in clinical practice, especially in treating relapsed or refractory leukemia patients.

#### 2.5.1. Monoclonal Antibodies

Monoclonal antibodies are laboratory-produced immune system proteins. Their application in leukemia is an important advancement in the field of leukemia treatment. Monoclonal antibodies can specifically bind to targets highly expressed on leukemia cells but sparsely expressed on normal cells. They exert effects through multiple mechanisms, including antibody-dependent cell-mediated cytotoxicity (ADCC), complement-dependent cytotoxicity (CDC), and directly inducing apoptosis [58]. Monoclonal antibodies can be used alone or in combination with chemotherapy drugs. Currently, multiple monoclonal antibodies have been applied in the treatment of leukemia patients.

##### Rituximab

**Figure 4:**
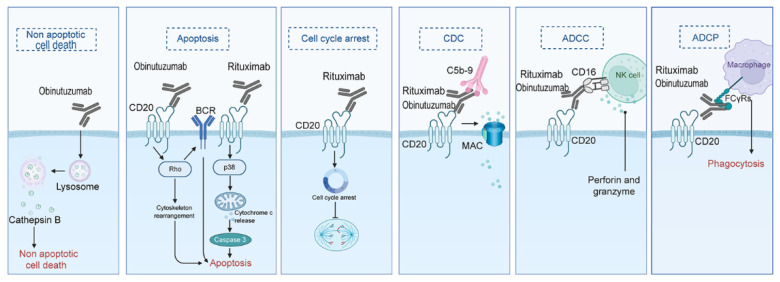
The Fab segment of Rituximab/Obinutuzumab specifically binds CD20 antigen, which leads to leukemia cell death through direct cytotoxic effects, antibody-dependent cell-mediated cytotoxicity, antibody-dependent cell phagocytosis, complement-dependent cytotoxicity, and induction of apoptosis. Among them, Obinutuzumab binds CD20 at a wider angle and has stronger direct cytotoxicity. Rituximab binds CD20 with lipid raft formation and strong CDC action, while Obinutuzumab binds CD20 without forming lipid rafts and thus has weaker CDC activity.

Rituximab (Figure 1) is an anti-CD20 immunotherapy drug, the first monoclonal antibody approved for cancer treatment, and received FDA approval in 1997 [59,60]. Research has found that Rituximab exerts effects on ALL, CLL, hairy cell leukemia (HCL), etc., through various mechanisms (Figure 4) [61,62,63]. Specific mechanisms include: inducing NK cell release of granzymes by binding to the Fc-γ receptor on NK cells, thereby leading to cell death [64,65]; binding to the CD20 molecule on the surface of B cells, activating the extrinsic apoptosis pathway to induce cell death [66]; binding to complement components (especially C1q), activating the complement system, forming the membrane attack complex, leading to cell death [67]; binding to the Fcγ receptor on macrophages, inducing immune cell phagocytosis and clearing B cells [68]. According to long-term follow-up results of Rituximab treatment for B-ALL, compared with the control group, the proportion of patients with minimal residual disease (MRD) level below 10^−4^ (i.e., less than 1 bone marrow blast in 10,000 normal cells) was significantly increased (91% vs. 82%); the 2-year EFS rate after a median follow-up of 30 months (65% vs. 52%), the cumulative relapse rate (18% vs. 32%), and the 4-year cumulative relapse rate (25% vs. 41%) all showed significant advantages; after induction therapy, the complete remission (CR) rate (92% vs. 90%) showed no significant difference between the two groups. In addition, the overall incidence of serious adverse events was similar between the two groups, but fewer patients in the Rituximab group experienced allergic reactions to asparaginase [61]. Overall, the results of this study indicate that Rituximab can be a new treatment option for B-ALL.

Rituximab can be used as monotherapy or in combination with other drugs for the treatment of HCL. However, when used as monotherapy, its efficacy for relapsed or refractory HCL patients is limited, rarely inducing complete remission [69]. In addition, it can cause infusion-related reactions and rarer toxicities such as cytopenia and infection [70]. Therefore, more effective drugs need to be developed to overcome these adverse reactions and improve therapeutic effects.

##### Obinutuzumab

Obinutuzumab (Figure 1) is a type II fully humanized anti-CD20 antibody. Compared with Rituximab, it has stronger antibody-dependent cell-mediated cytotoxicity and direct cell death effects, but weaker complement activation effects (Figure 4) [71]. It is currently approved for CLL. According to the GAUGUIN study, Obinutuzumab monotherapy is safe and effective in R/R-CLL patients, especially in patients with low baseline tumor burden. The Phase I trial showed a good dose-response relationship, with an overall response rate and best overall response rate both being 62%. In the Phase II trial, the overall response rate was 15%, and the best overall response rate was 30%. The lower response rate may be related to the higher baseline tumor burden of the patients [72].

Compared with Rituximab, although Obinutuzumab shows better efficacy in CLL, the incidence of grade 3–4 adverse events is significantly increased, especially in terms of infusion-related reactions, thrombocytopenia, and cardiac events. In addition, the rates of grade 3–4 infection and drug discontinuation also tend to increase [70].

#### 2.5.2. Bispecific T-Cell Engager

Bispecific T-cell Engager (BiTE) is a bispecific antibody capable of simultaneously binding to tumor-associated antigens and the CD3 molecule on the surface of T cells [73]. BiTEs consist of two single-chain variable fragments (scFv) connected by a flexible linker peptide. One scFv specifically binds to tumor-associated antigens on the surface of tumor cells, such as CD19, BCMA, etc.; the other scFv binds to the CD3 molecule on the surface of T cells [74]. As a new class of immunotherapy drugs, it has shown significant application value in the treatment of relapsed/refractory R/R-ALL.

CD19/CD3 bispecific antibody is a novel immunotherapy drug mainly used for treating B-cell related hematological malignancies, such as ALL. This antibody can simultaneously specifically bind to the CD19 antigen on the surface of B cells and the CD3 antigen on the surface of T cells, mediating the killing effect of T cells on CD19-expressing malignant B cells. Currently, this type of antibody has shown good therapeutic effects and safety in clinical trials. As an FDA-approved CD19/CD3 bispecific antibody, it is currently used for the treatment of B-ALL [75,76].

**Figure 5:**
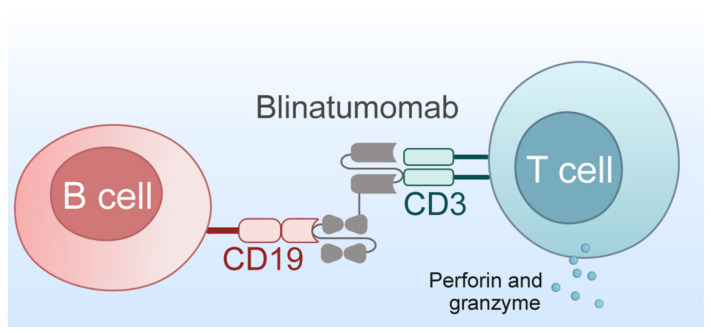
Blinatumomab creates an anti-leukemic effect by targeting CD3 and CD19 to establish a link between T cells and tumor B cells, inducing T cells to release granzyme and perforin to target and lyse tumor cells.

Blinatumomab (Figure 1) is a novel BiTE that can simultaneously bind to CD3 on T cells and CD19 on the surface of B-ALL cells, thereby activating T cells to kill tumor cells (Figure 5) [76]. It was approved by the FDA in 2014 and approved by China’s NMPA in 2020. It is the first approved standard BiTE molecule and the only approved drug for measuring MRD in ALL treatment. It is mainly used for treating adult relapsed/refractory precursor B-ALL [77]. Blinatumomab can be used as monotherapy or in combination with chemotherapy drugs for the treatment of B-ALL. Results of a phase 3 clinical trial showed that compared with standard chemotherapy, Blinatumomab treatment for adult relapsed/refractory precursor B-ALL has significant advantages. Comparative analysis revealed that the Blinatumomab group significantly prolonged OS and DOR, with a median OS of 7.7 months (chemotherapy group 4.0 months), reducing the risk of death by 29%. In addition, the CR rate (including complete remission with full hematologic recovery) (34% vs. 16%) and CRi rate (complete remission with incomplete or partial hematologic recovery) (44% vs. 25%) in the Blinatumomab group, as well as the 6-month EFS (31% vs. 12%), were significantly superior to the chemotherapy group. Although the Blinatumomab group reported higher rates of neurological events and cytokine release syndrome, the overall incidence of adverse events was lower. This study provides new hope for the treatment of adult relapsed or refractory B-cell precursor ALL, especially in cases where traditional chemotherapy effects are limited [76].

### 2.6. Differentiation Inducers

Differentiation inducers are a special type of drug that inhibits the unlimited proliferation of abnormal or immature cells and may eventually lead to their death by promoting them to regain normal maturation and differentiation ability. Among them, all-trans retinoic acid (ATRA) is a representative differentiation inducer. Its application in the treatment of acute promyelocytic leukemia (APL) has effectively changed the treatment strategy of the disease, transforming this once extremely lethal cancer into a highly curable disease [78].

As a retinoic acid differentiation inducer, ATRA is one of the earliest drugs used in tumor differentiation induction therapy and is now widely recognized as the first choice for APL induction therapy. Its mechanism of action is mainly through binding to the PML-RARα fusion protein unique to APL, relieving its inhibitory effect on differentiation-related genes, thereby inducing abnormal promyelocytes to differentiate into mature neutrophils [79]. However, long-term use may cause some adverse reactions, such as increasing the risk of differentiation syndrome, inducing excessive inflammation, a higher risk of relapse, and possible hyperleukocytosis [80].

### 2.7. Upcoming Targeted Therapies Under Trial

Currently, many new targeted therapeutic drugs are in the clinical trial stage, and some of them have shown good efficacy. For example, Ziftomenib, an oral selective menin inhibitor developed for patients with relapsed or refractory AML with NPM1 mutations and KMT2A rearrangements. Its mechanism of action is mainly to disrupt the binding of Menin to the binding pocket in MLL1/2 and MLL1-FP, thereby reducing the binding of MLL1/2 and MLL1-FP to their targets, inhibiting HOXA9/MEIS1 activity, and inducing differentiation and loss of viability of AML with MLL1-r or mutant (mt)-NPM1 [81]. According to the results of the KOMET-001 trial, 24% of patients achieved complete remission or complete remission with partial hematological recovery, and most adverse events are currently manageable [82]. These results show that Ziftomenib, as a new oral selective menin inhibitor, has potential clinical application value in the treatment of relapsed or refractory AML.

Nemtabrutinib, an oral reversible BTK inhibitor, can effectively inhibit C481S mutant BTK and activated PLCγ2, overcoming the drug resistance after ibrutinib treatment [83]. According to the results of a phase I clinical trial, the overall remission rate for CLL patients receiving 65 mg daily treatment reached 75% [84]. This shows that Nemtabrutinib has significant potential in the treatment of CLL.

### 2.8. Limitations of Targeted Therapy

In recent years, the field of targeted therapy has developed rapidly, especially in the research and development of new drugs targeting specific molecular targets and the optimization of combination therapy strategies. Taking CML as an example, TKIs such as Imatinib (first-generation) and Dasatinib (second-generation) have significantly improved patient survival rates and offer the possibility of achieving “treatment-free remission” [16,22]. Although targeted therapy effects are significant, monotherapy use has limitations. First, resistance is a major problem. In CML treatment, as time progresses, some patients develop resistance to TKIs, leading to disease progression. Research shows that about 25% of patients develop resistance after Imatinib treatment [85]. In AML, resistance often occurs after FLT3 inhibitor treatment, mainly due to secondary mutations in the FLT3 kinase domain, which prevent the drug from effectively binding to the target, reducing treatment efficacy [42].

Secondly, monotherapy is difficult to completely eradicate leukemia cells. Leukemia cells exhibit high heterogeneity, with different cell subpopulations possibly relying on multiple signaling pathways for survival. A single targeted drug may only inhibit the growth of some cells and cannot completely eliminate all leukemia cells. In addition, monotherapy may not overcome the protective effect of the tumor microenvironment on leukemia cells. Cytokines, stromal cells, etc., in the tumor microenvironment can promote the survival and proliferation of leukemia cells, reducing the efficacy of monotherapy. Moreover, monotherapy may cause some adverse reactions, affecting the patient’s quality of life and treatment compliance, limiting the dosage and duration of drug use, thus affecting treatment efficacy. To address these issues, combination therapy has become a new research direction. Combination therapy can improve treatment efficacy, overcome resistance, and reduce adverse reactions through the synergistic effects of different drugs.

## 3. Combination Therapy

In the treatment of leukemia, the overall efficacy of monotherapy with targeted drugs is not ideal. To effectively address key issues such as drug resistance and relapse, combination therapy strategies have gradually received attention. For example, the combined application of Venetoclax and hypomethylating agents has a significantly higher overall complete remission rate than the use of Azacitidine alone [86]. Therefore, combination therapy has become a hot research direction in the field of leukemia treatment. The combination application modes of targeted drugs are diverse, including the combination of targeted drugs and chemotherapy drugs, sequential therapy between targeted drugs, synergistic application of targeted drugs and CAR-T cell therapy, and multi-drug combination schemes, etc.

### 3.1. Combination of Targeted Drugs and Chemotherapy Drugs

Given the certain limitations of monotherapy targeted therapy in leukemia treatment, many studies in recent years have focused on the combined application of targeted drugs and chemotherapy drugs in leukemia treatment to enhance its therapeutic effect (Table 1).

Taking Ph+ALL and AML as examples, studies have found that the combination therapy of targeted drugs and chemotherapy drugs can significantly improve the survival rate of leukemia patients and effectively improve their prognosis.

For Ph+ALL patients, relevant studies have shown that when using Imatinib combined with a low-intensity CVAD treatment plan, the patient’s 5-year OS rate reached 45.6%, and EFS was 37.1% [14]. Compared with traditional chemotherapy plans, adding Imatinib significantly improved the patient’s complete remission rate and long-term survival rate. In addition, the latest results of the Ph+ ALL CON trial show that Ponatinib combined with low-intensity chemotherapy for newly diagnosed Ph+ALL adult patients is superior to Imatinib combined with low-intensity chemotherapy. At the end of induction therapy, the MRD-negative complete remission rate in the Ponatinib group was significantly higher than in the Imatinib group (34.4% vs. 16.7%), and the safety of the two treatment plans was comparable [87].

Furthermore, the combination of targeted drugs and the Hyper-CVAD regimen has better therapeutic effects for high-risk Ph+ALL patients, but attention should be paid to drug dosage. For example, using Ponatinib combined with the Hyper-CVAD regimen to treat Ph+ALL patients, the CR reached 100%, the complete molecular remission rate was 86%, and the 6-year OS rate was 75%. In addition, most patients did not need HSCT after achieving complete remission for the first time, and after dose adjustment, the safety of this treatment strategy was significantly improved [88]. Results from another phase II clinical study showed that the Hyper-CVAD combined with a sequential Blinatumomab treatment plan has significant efficacy for Ph-negative B-cell ALL adult patients. This treatment plan significantly improved the patient’s 3-year OS rate (81%) and relapse-free survival rate (73%), and during the treatment process, the MRD negativity rate was as high as 97% [75].

For AML patients, the combined application of targeted drugs and chemotherapy drugs has also achieved significant results. Whether in AML patients of all age groups or in AML patients carrying FLT3 mutations, this combination therapy method has shown good efficacy. For example, for elderly AML patients who are untreated and unsuitable for intensive chemotherapy, compared with Azacitidine alone, the combined use of Venetoclax and Azacitidine can significantly prolong the patient’s overall survival and improve the overall complete remission rate. Although the incidence of febrile neutropenia is relatively high in the combination therapy group, overall, the safety and effectiveness of this combination therapy are superior to Azacitidine alone [86]. Currently, this combination therapy plan has been identified as the standard treatment method for elderly or medically unfit AML patients who cannot tolerate intensive chemotherapy. There are also studies combining Venetoclax with FLAG-IDA for the treatment of newly diagnosed AML or R/R-AML patients. The ORR of this combination plan is 98%, and the MRD-negative complete remission rate is 93%. The 24-month OS rate is 76%, and EFS is 64%, and the adverse events are similar to traditional chemotherapy and manageable. In addition, this study also emphasized the key role of TP53 mutation in predicting treatment outcome and survival rate, providing an important direction for future treatment of this high-risk subgroup [89].

In addition, the RATIFY study results showed that Midostaurin combined with chemotherapy drugs has significant efficacy for AML patients carrying FLT3 mutations. Compared with the placebo group, the Midostaurin group significantly prolonged OS (74.7 months vs. 25.6 months) and EFS (8.2 months vs. 3.0 months), but there was no significant difference in CR (58.9% vs. 53.5%), and the incidence of adverse events was similar to the placebo group, indicating that Midostaurin combined chemotherapy plan can significantly improve the survival prognosis of AML patients carrying FLT3 mutations [90]. This conclusion was further validated in a phase IIIb clinical trial. Midostaurin combined with chemotherapy has safe and high response rate treatment options for adult FLT3-mutated AML patients of all age groups. This drug has now been approved for use in combination with standard induction and consolidation chemotherapy for the treatment of newly diagnosed FLT3 AML adult patients [91].

In summary, combining traditional chemotherapy drugs with targeted drugs effectively improves the OS and CR rates of leukemia patients. Currently, some combination therapy plans have been used as standard treatment plans for leukemia. However, it also faces problems such as drug interactions and complex treatment plans, which need continuous optimization and adjustment in clinical practice.

### 3.2. Internal Combination of Targeted Drugs

#### 3.2.1. Internal Combination of Inhibitors

Based on the significant monotherapy activity of inhibitors in leukemia treatment, and the significant improvement in treatment effects when they are used in internal combination, researching new combination strategies (Table 2) to inhibit leukemia cells through synergistic effects, thereby improving efficacy, holds important significance.

Taking CML as an example, numerous studies have explored the combined application of targeted drug inhibitors. For instance, a phase 2 clinical trial showed that alternating Nilotinib and Imatinib treatment might help improve efficacy, enhance safety, and reduce costs. Study results indicated that the 5-year PFS and OS rates for this regimen were both 89%, and its safety, especially regarding cardiovascular adverse events, was superior to Nilotinib monotherapy [99]. There are also studies combining Dasatinib with Venetoclax for the treatment of newly diagnosed chronic myeloid leukemia in chronic phase (CML-CP) patients. Research found that the combination therapy of Dasatinib and Venetoclax is safe, feasible, and effective. At 12 months of treatment, MMR was 86%, and DMR was 53%. However, compared to Dasatinib monotherapy, the combination therapy did not significantly improve MMR and DMR rates, but instead led to a higher incidence of neutropenia [100].

Furthermore, several targeted drug inhibitor combination regimens currently in the research phase have shown good effects on CML resistance and synergy. For example, Mariarita Spampinato et al. found that combining Dasatinib with Selinexor to treat human CML cell lines (LAMA84 and K562) exhibited significant inhibitory effects. Its mechanism may involve impairing mitochondrial function and downregulating the nuclear translocation of HO-1, increasing the sensitivity of leukemia stem cells (LSCs) to apoptosis, reducing their ability to maintain the disease state, and thus potentially overcoming drug resistance [101]. Additionally, studies have shown that the combination of Imatinib and Carfilzomib exhibits synergistic effects in the treatment of CML [102].

In summary, the combined application of targeted drug inhibitors shows better efficacy in leukemia treatment, especially in overcoming drug resistance and improving treatment efficiency. However, some combination regimens perform poorly in terms of safety. Therefore, further exploration of new combination therapy strategies and optimization of drug combinations are needed to provide patients with more effective treatment options.

#### 3.2.2. Combination of Inhibitors and Immunotherapy Drugs

By combining inhibitors with immunotherapy drugs, multiple targets of leukemia cells can be targeted simultaneously, thereby enhancing therapeutic effects, reducing drug resistance, and decreasing the occurrence of side effects. Currently, various combination regimens of inhibitors and immunotherapy drugs have been developed (Table 3).

In CLL treatment, a long-term follow-up study showed that the PFS and OS rates of CLL patients treated with Obinutuzumab combined with Chlorambucil (O+Chl) were significantly better than those treated with Rituximab combined with Chlorambucil (R+Chl). Especially in terms of OS, the O+Chl group showed a clear survival advantage [103].

However, the CLL14 study found that a fixed-duration regimen of Venetoclax plus Obinutuzumab showed significantly better long-term efficacy and safety in treatment-naive CLL patients compared to Obinutuzumab plus Chlorambucil. Results showed that the 6-year OS rate of the V+O group was significantly higher than the O+Chl group (78.7% vs. 69.2%), and the V+O group had significantly longer PFS and time to next treatment, with significantly improved quality of life [104].

Similarly, another study, ELEVATE-TN, showed that Acalabrutinib combined with Obinutuzumab (A+O) demonstrated better safety and tolerability compared to the O+Chl regimen. The results of this study showed that after a median follow-up of 74.5 months, the median PFS of the A+O combination therapy was not reached, while the median PFS of O+Chl was 27.8 months. Furthermore, the 72-month survival rate of the A+O group was 83.9%, significantly better than the A group and the O+Chl group (75.5% and 74.7%) [105]. This indicates significant individual differences in patient treatment.

Additionally, studies have used Rituximab combined with Ibrutinib (IR) for CLL treatment. According to the interim analysis of the FLAIR trial, compared with the Fludarabine, Cyclophosphamide, Rituximab (FCR) combination regimen, after a median follow-up of 53 months, the median PFS of the IR group was not reached, significantly superior to the median PFS of 67 months in the FCR group. However, the 4-year OS rates (92.1% vs. 93.5%) showed no significant difference, possibly because patients received effective second-line targeted therapy after disease progression. But the IR group treatment increased the risk of cardiovascular events, especially in patients already receiving treatment for hypertension or cardiovascular disease. Therefore, patients can choose the corresponding treatment plan based on their condition. If using the IR group treatment, a comprehensive cardiac evaluation should be performed first [62].

Overall, the combined application of inhibitors and immunotherapy drugs shows significant efficacy in the treatment of leukemia, especially in improving PFS and OS rates. However, the safety and tolerability of different combination regimens vary, and individual patient differences significantly impact treatment outcomes. Therefore, during the treatment process, the most suitable treatment plan should be selected based on the specific situation of the patient, and a comprehensive evaluation should be conducted before treatment to ensure the safety and effectiveness of the treatment. At the same time, further development of new combination therapy strategies is needed to meet the needs of different patients.

**Table 3:** Internal Combination of Targeted Drugs.

Combination Therapy Type	Combined Drugs	Indication	Patient Number	Treatment Outcome	Reference
Inhibitor Internal Combination	Imatinib, Nilotinib	CML	123	5-year OS and PFS rates both 89%	[99]
	Dasatinib, Venetoclax	CML	65	4-year EFS and OS rates 96% and 100% respectively	[100]
	Dasatinib, Selinexor	CML	None	Produces significant inhibitory effects on LAMA84 and K562 cell lines	[101]
	Imatinib, Carfilzomib	CML	None	Can significantly reduce proliferation and induce CML stem cell apoptosis	[102]
	Ponatinib, Asciminib	CML	None	Effectively overcomes resistance caused by BCR-ABL1 compound mutations	[106]
	Imatinib, Venetoclax	CLL, CML	1	Achieved DMR	[107]
	Acalabrutinib, Venetoclax	CLL	867	36-month OS rate 94.1%	[108]
	Idelalisib, Tirabrutinib	CLL	53	Objective response rate 93%, CR 7%	[109]
	Gilteritinib, MEN1703	AML	None	Simultaneously inhibits PIM and FLT3, significantly enhances anti-tumor activity in vivo/in vitro	[110]
	Gilteritinib, GSK-J4	AML	None	Combination therapy of Gilteritinib and GSK-J4 can significantly inhibit the growth of MV4-11 and MOLM-13 cells	[111]
Inhibitors in combination with immunologic drugs	Acalabrutinib, Obinutuzumab	CLL	535	72-month OS rate 83.9%, PFS rate 78%	[105]
	Obinutuzumab, chlorambucil	CLL	35	8-year OS rate 61%, EFS rate 25%	[103]
	Obinutuzumab, Venetoclax	CLL	432	6-year OS rate 78.7%, for patients with del(17p) and/or TP53 mutation, 6-year OS rate 60.0%	[104]
	Ibrutinib, Ublituximab	CLL	126	Median follow-up 41.6 months, ORR 83%	[112]
	Rituximab, Idelalisib	CLL	110	ORR 83.6%	[45]
	Rituximab, Ibrutinib	CLL	1924	4-year OS rate 92.1%	[62]
	Rituximab, Venetoclax	CLL	389	2-year OS rate 91.9%	[113]
	Dasatinib, Blinatumomab	Ph+ ALL	63	Median follow-up 18 months, OS rate 95%, Disease-Free Survival 88%	[114]
	Rituximab, Vemurafenib	HCL	30	Median follow-up 34 months, Relapse-Free Survival rate 85%	[63]

### 3.3. Combination of Targeted Drugs and CAR-T

CAR-T is a revolutionary cancer treatment method that modifies a patient’s own T cells to enable them to more effectively recognize and attack cancer cells in the body. The specific process involves extracting T cells from the patient’s body, genetically modifying these cells to produce chimeric antigen receptors on their surface that target specific cancer cell antigens. These modified T cells are called CAR-T cells, which are then expanded and reinfused into the patient’s body with the aim of eliminating cancer cells [115,116]. Currently, anti-CD19 CAR-T therapy targeting malignant B cells in leukemia has received FDA and EMA approval and has shown good efficacy in clinical applications [117].

Although CAR-T cell therapy has achieved significant success in B-ALL, treatment failure due to disease relapse or primary resistance, as well as serious side effects such as cytokine release syndrome and immune effector cell-associated neurotoxicity syndrome, still limit the widespread application of CAR-T cell therapy [118]. Therefore, it is necessary to reduce its toxic side effects and increase treatment efficacy through combination therapy. A prospective single-center phase II clinical trial showed that combining Ibrutinib and CAR-T cell therapy can achieve higher CR and longer PFS in CLL patients who have not achieved complete remission. At 3 months, the complete remission rate was 43.8%, at 12 months, it was 50%, and at 12 months, 72.2% of patients had undetectable MRD. At 48 months, the overall survival rate was 84%, and the progression-free survival rate was 70%. This indicates that combination therapy has high safety and effectiveness in CLL and can significantly improve patient prognosis [119].

Furthermore, the latest research shows that Dasatinib combined with CAR-T cells for the treatment of newly diagnosed Ph+AML adult patients shows a complete molecular remission rate reaching 85%, and the 2-year OS rate and leukemia-free survival rate are both 92%, with good safety and efficacy [120].

Multiple studies (Table 4) have demonstrated that the combination of targeted drugs and CAR-T has good efficacy in the treatment of leukemia. Although there are still certain limitations, new CAR-T combination methods can be continuously developed subsequently to effectively improve the cure rate of leukemia.

### 3.4. Multi-Drug Combination

Although combination therapy for leukemia shows significant advantages in improving efficacy, problems still exist in terms of safety and tolerability. By using multiple drugs with different mechanisms of action in combination, leukemia cells can be attacked from multiple angles, showing more obvious advantages in overcoming drug resistance, reducing side effects, improving survival rates, and prolonging disease-free survival. Currently, various multi-drug combination therapies for leukemia have been developed and applied (Table 5).

For example, in the treatment of CLL, the iFCG regimen showed high efficacy and low side effects in newly diagnosed CLL patients. Results of a phase 2 clinical trial showed that 87% of patients achieved undetectable MRD (U-MRD) after 3 cycles of chemotherapy, and 98% of patients achieved the best U-MRD response after 12 cycles. The 3-year PFS rate and OS rate were both 98% [123]. This study provides a new, time-limited chemoimmunotherapy (CIT) option for CLL patients, capable of improving U-MRD rates while reducing the burden of chemotherapy, potentially achieving longer disease-free survival and lower long-term side effect risks, especially suitable for young patients in good physical condition.

For relapsed CLL patients, the triple combination therapy of Tirabrutinib, Idelalisib, and Obinutuzumab (TIO group) showed higher efficiency and better safety compared to the combination of Tirabrutinib and Idelalisib (TI group). Results showed that compared to the TI group, at week 25, the ORR of the TIO group was (93.3% vs. 60.0%), and the 24-month PFS was also higher (80.6% vs. 60.0%). In addition, although the CR rate of the TIO group at week 25 was only 6.7%, during subsequent follow-up, the U-MRD negativity rate of the TIO group significantly increased, from 6.7% to 36.7% [124].

Furthermore, the latest research found that the triple-drug combination therapy of Zanubrutinib, Venetoclax, and Obinutuzumab, based on MRD detection, shows higher efficacy in R/R-CLL patients. After 6 cycles of induction therapy, 52.5% of patients achieved peripheral blood U-MRD, and the best U-MRD rate reached 85%. The 18-month OS rate was 96%, and the PFS rate was 96.8%. Although there were more COVID-19-related adverse events, the overall safety was good, and no new unknown resistance mutations appeared [125].

In summary, multi-drug combination therapy for leukemia shows significant advantages in improving treatment efficacy, reducing toxic side effects, prolonging survival, and improving quality of life. By optimizing treatment plans, adverse reactions during the treatment process are significantly reduced, and the patient’s quality of life is significantly improved. However, the long-term survival status is still unsatisfactory, requiring continuous development of new drug combinations and research into new treatment methods.

## 4. Conclusions and Outlook

Over the past several decades, substantial advancements have been achieved in the pharmacological management of leukemia. Nevertheless, chemotherapy, as the primary treatment modality, continues to exhibit certain limitations. Firstly, traditional chemotherapeutic agents operate by targeting rapidly dividing cells, yet they lack sufficient specificity. This often results in significant toxicity and an increased risk of secondary infections, hemorrhage, organ damage, and secondary tumor development [131,132]. Secondly, chemotherapeutic agents are susceptible to inducing drug resistance during their application, and certain leukemias demonstrate insensitivity to these drugs. Consequently, the efficacy of chemotherapy as a standalone treatment is often suboptimal. Even when CR is attained, residual LSCs may persist within the patient’s body. These LSCs remain in a quiescent state and exhibit insensitivity to chemotherapy, thereby constituting the underlying cause of MRD and subsequent relapse [133].

The advent of targeted therapies has significantly enhanced the prognosis for patients with leukemia, offering renewed optimism for treatment. These therapies function by selectively binding to specific targets, thereby effectively inhibiting the growth and proliferation of leukemia cells while minimizing harm to normal cells. Imatinib, the first kinase inhibitor, exemplifies this advancement by revolutionizing CML treatment, transforming it from a rapidly fatal disease into a manageable condition [134]. However, long-term monotherapy presents several challenges, including drug relapse and resistance. Consequently, the development of novel therapeutic regimens is imperative to improve OS and PFS rates in leukemia, with the ultimate goal of achieving a cure.

Consequently, combination therapies have been formulated to achieve profound and sustained remission by leveraging the synergistic effects of multiple pharmacological agents, thereby extending the duration of treatment-free intervals and markedly enhancing the therapeutic efficacy in leukemia management. For instance, the concurrent administration of Rituximab and Vemurafenib in the treatment of relapsed HCL has been shown to significantly elevate the complete remission rate and reduce the treatment duration. Additionally, this combination effectively improves the MRD clearance rate, thereby prolonging relapse-free survival and facilitating rapid, safe, and durable complete remission [63].

While combination therapy has yielded significant outcomes, it continues to encounter numerous challenges, with relapse being a predominant issue in the current treatment paradigm. For instance, in the treatment of core binding factor acute leukemia (CBFL) using Midostaurin in conjunction with intensive chemotherapy, 97% of patients attained complete remission; however, 51.51% experienced relapse within two years of achieving remission [95]. Therefore, the synergistic application of combination therapy alongside targeted agents requires further refinement to optimize leukemia treatment outcomes.

Furthermore, a substantial challenge in combination therapy lies in the variability among individual patients. Responses to combination therapy can vary significantly, as patients exhibit diverse biological behaviors and drug sensitivities across different types and stages of leukemia. Consequently, when devising a combination therapy regimen, it is imperative to thoroughly account for patient-specific variability. This involves selecting the most appropriate drug combinations, dosages, and treatment sequences through a comprehensive evaluation of each patient. Such an approach aims to enhance the efficacy and safety of combination therapy, thereby advancing the realization of precision medicine [135,136].

In the future, with the help of advanced technologies such as high-throughput sequencing, single-cell sequencing, metabolomics, etc., we will comprehensively analyze the molecular characteristics of different leukemia cells, reveal the abnormal genes, protein expression and their interaction networks, further analyze in depth the pathogenesis of leukemia and the drug resistance mechanism, and search for new therapeutic targets and research and development of new targeted drugs. For example, the development of inhibitors for PI3Kγ, a newly discovered therapeutic target, will provide a new strategy for the combination therapy of leukemia [137].

In conclusion, the continuous development of combination therapy will bring better therapeutic prospects for leukemia patients and is expected to further improve the cure rate of leukemia and the long-term survival rate of patients.

## Figures and Tables

**Table 1:** Representative drugs for targeted therapy.

Combination Therapy Type	Combined Drugs	Indication	Patient Number	Treatment Outcome	Reference
Tyrosine kinase inhibitors	Imatinib	Ph+ALL, CML	1106	10-year OS rate 83.3%	[22]
	Dasatinib		149	5-year OS rate 96%, treatment failure-free survival rate 95%.	[16]
	Ponatinib		51	10-year OS rate 90%, 2-year EFS 97%	[32]
FLT3 Inhibitors	Midostaurin	AML	22	overall response rate was 55.5%, OS 3.7 months.	[38]
	Gilteritinib		247	26 patients survived for 2 years or longer without recurrence	[41]
B-Cell Signaling Pathway Inhibitors	Ibrutinib	CLL	269	ORR 92%, PFS 70%, OS rate 83%.	[46]
	Acalabrutinib		134	45 months PFS 62%	[50]
	Idelalisib		54	81.5% of patients achieved lymph node response during treatment	[51]
Anti-apoptotic Inhibitors	Venetoclax	CLL and AML	-	-	-
Immunotherapy Drugs	Rituximab	ALL, CLL, HCL	209	2-year EFS 65%	[61]
	Obinutuzumab		33	OS rate 62%, best overall response rate 62%	[72]
	Blinatumomab		405	Median OS 7.7 months, CR rate was 34%.	[76]
Differentiation Inducers	ATRA	APL	-	-	-
up coming targeted therapies under trial	Ziftomenib	AML	83	25% achieved complete remission or complete remission with partial hematologic recovery	[82]
	Nemtabrutinib	CLL	48	OS rate in patients with CLL was 75%.	[84]

**Table 2:** Targeted Drugs + Chemotherapy Drugs.

Combination Therapy Type	Combined Drugs	Indication	Patient Number	Treatment Outcome	Reference
TKI + Chemotherapy Drugs	Imatinib, CVAD (Cyclophosphamide/Vincristine/Doxorubicin/Dexamethasone)	Ph+ ALL	268	5-year OS rate 45.6%, EFS rate 37.1%	[14]
	Ponatinib, low-intensity chemotherapy regimen	Ph+ ALL	245	MRD 34.4%	[87]
	Ponatinib, Hyper-CVAD (Cyclophosphamide/Vincristine/Doxorubicin/Dexamethasone)	Ph+ ALL	86	6-year OS rate 75%	[88]
	Dasatinib, Hyper-CVAD (Cyclophosphamide/Vincristine/Doxorubicin/Dexamethasone)	CML-LBP, Ph+ ALL	85	CML-LBP and Ph+ ALL 5-year OS rates were 59% and 48%, respectively	[92]
	Dasatinib, Decitabine	CML	30	Median OS of 13.8 months	[93]
	Dasatinib, Cytarabine, Daunorubicin	CBF-AML	61	3-year Disease-Free Survival and OS 75% and 77% respectively	[94]
Anti-apoptotic inhibitors + chemotherapeutic agents	Venetoclax, Azacitidine	AML	431	Median follow-up 20.5 months, OS 14.7 months	[86]
	Venetoclax, FLAG-IDA (Fludarabine, Cytarabine, Granulocyte Colony-Stimulating Factor, Idarubicin)	AML	45	ORR 98%, MRD 93%, 24-month OS rate 76%, EFS 64%	[89]
B-cell signaling pathway inhibitors + chemotherapeutic agents	Midostaurin, Daunorubicin, Cytarabine	AML	717	Median OS 74.7 months	[90]
	Midostaurin, Daunorubicin or Idarubicin	AML	301	CR+CRi 80.7%, of which 65.3% reached CR	[91]
	Midostaurin, Cytarabine, Daunorubicin or Idarubicin	Core binding factor leukemia (CBFL)	34	Median follow-up 31.5 months, OS rate 73.52%	[95]
	Midostaurin, intensive chemotherapy	AML	440	2-year OS rate 55%, EFS rate 41%, CR/CRi 74.9%	[96]
	Gilteritinib, Mitoxantrone	AML	none	Significantly inhibited the proliferation of MV4-11 and MOLM13 cells	[35]
	Gilteritinib, intensive induction and consolidation chemotherapy	AML	103	Median overall survival time 46.1 months	[97]
Immunotherapeutic drugs + chemotherapeutic drugs	Blinatumomab, Hyper-CVAD (Cyclophosphamide/Vincristine/Doxorubicin/Dexamethasone)	B-ALL	38	3-year OS rate 81%	[75]
	Rituximab, Fludarabine, Cyclophosphamide	CLL	1924	4-year OS rate 93.5%	[62]
	Obinutuzumab, Fludarabine, Cyclophosphamide	CLL	630	4-year OS rate in G-FC group reached 90%+	[98]

**Table 4:** Targeted Drugs + CAR-T Combination.

Combination Therapy Type	Combined Drugs	Indication	Patient Number	Treatment Outcome
CAR-T, Ibrutinib	CLL	20	At 48 months, OS rate 84%, progression-free survival rate 70%	[119]
CAR-T, Dasatinib	Ph+ AML	28	2-year OS and leukemia-free survival rates both 92%	[120]
CAR-T, Gilteritinib	AML	None	Significantly enhanced the anti-AML effect of CAR T cells	[121]
CAR-T, Duvelisib	CLL	None	Significantly improved the survival rate of CLL mice	[122]

**Table 5:** Multi-drug combinations.

Combination Therapy Type	Combined Drugs	Indication	Patient Number	Treatment Outcome
Ibrutinib, Fludarabine, Cyclophosphamide, Obinutuzumab	CLL	45	3-year OS rate 98%	[123]
Idelalisib, Ttirabrutinib, Obinutuzumab	CLL	35	ORR 93.3%	[124]
Zanubrutinib, Venetoclax, Obinutuzumab	CLL	42	18-month OS rate 96.8%, PFS rate 96%	[125]
Ibrutinib, Venetoclax, Obinutuzumab	CLL	50	2 months after end of treatment, CR 28%	[126]
Gilteritinib, Azacitidine, Venetoclax	AML	52	18-month OS rate 72%	[127]
Dasatinib, Asciminib, Prednisone	Ph+ ALL	24	2-year OS rate 75%	[128]
Dasatinib, Prednisone, Blinatumomab	Ph+ ALL	24	3-year OS rate 87%	[129]
Ponatinib, Decitabine, Venetoclax	CML	20	1-year and 2-year OS rates 41% and 34% respectively	[130]
Acalabrutinib, Venetoclax, Obinutuzumab	CLL	867	36-month OS rate 87.7%	[108]

Combination Therapy Type

Combined Drugs

Indication

Patient Number

Treatment Outcome

## Abbreviations

ALL	acute lymphoblastic leukemia;
AML	acute myeloid leukemia;
CLL	chronic lymphocytic leukemia;
CML	chronic myeloid leukemia;
TKIs	Tyrosine kinase inhibitors;
CAR	T-Chimeric Antigen Receptor T-Cell;
Ph+ ALL	Philadelphia positive acute lymphoblastic leukemia;
HSCT	Hematopoietic Stem Cell Transplantation;
DMR	Deep Molecular Response;
TFR	Treatment-free remission;
OS	Overall Survival;
MMR	Major Molecular Response;
EFS	event-free survival;
FLT3-ITD	FLT3 internal tandem duplication mutations;
BTK	Bruton’s tyrosine kinase;
BCR	B-cell receptor;
ORR	Objective Response Rate;
PFS	progression-free survival;
R/R-CLL	relapsed/refractory chronic lymphocytic leukemia
BCL-2	B-cell lymphoma-2;
BiTE	bispecific T-cell engager;
HCL	hairy cell leukemia;
MRD	minimal residual disease;
CR	Complete Remission;
scFv	single-chain variable fragments;
R/R-ALL	relapsed/refractory acute lymphoblastic leukemia;
CML-CP	Chronic Phase CML;
LSCs	leukemia stem cells;
U-MRD	undetectable Minimal Residual Disease
CRi	Complete Remission with Incomplete Blood Count Recovery;
ATRA	all-trans retinoic acid;
APL	acute promyelocytic leukemia.
